# Methomyl resistance in *Musca domestica*: mechanisms and inheritance

**DOI:** 10.7717/peerj.21646

**Published:** 2026-08-24

**Authors:** Hafiz Azhar Ali Khan

**Affiliations:** Department of Entomology, University of the Punjab, Lahore, Punjab, Pakistan

**Keywords:** Methomyl, Houseflies, Insecticide resistance, Resistance management, Mode of inheritance

## Abstract

Methomyl resistance is a documented problem in *Musca domestica* field strains worldwide, including Pakistan, where the insecticide is commonly used. To address this, a methomyl-resistant near-isogenic line (Meth-R) was created specifically to determine its resistance inheritance mode and cross-resistance profile. Analysis of reciprocal F_1_ and F_1’_ crosses demonstrated autosomal inheritance of methomyl resistance in the Meth-R strain, as evidenced by similar median lethal dose (LD_50_) values and the absence of sex linkage or maternal effects. Resistance expression was incompletely dominant, with dominance values of 0.7 (F_1_) and 0.6 (F_1’_). Significant deviations between observed and expected mortality in self-bred (F_2_ and F_2’_) and backcross (BC_1_-BC_4_) progenies (chi-square analyses) indicate resistance is likely controlled by multiple genes. The Meth-R strain showed no cross-resistance to alpha-cypermethrin, fluralaner, pirimiphos-methyl, or clothianidin. Additionally, observing significant synergism of methomyl with both piperonyl butoxide (PBO; oxidase inhibitor) and *S,S,S*-tributyl phosphorotrithioate (DEF; esterase inhibitor) implicates metabolic detoxification as a key mechanism of methomyl resistance. The Meth-R strain exhibits methomyl resistance inherited as an autosomal, incompletely dominant trait under polygenic control. Although there was cross-resistance in the Meth-SEL strain to alpha-cypermethrin, fluralaner, pirimiphos-methyl, and clothianidin versus the lab susceptible strain, selection with methomyl did not increase the level of cross-resistance. These results offer a foundation for combating methomyl resistance.

## Introduction

Intensive and sustained insecticide application over the past decades has led to significant challenges in pest management (Pretty & Bharucha, 2015). The overuse and misuse of these chemicals have resulted in the evolution of resistance, environmental contamination, and adverse impacts on non-target species, underscoring the imperative for integrated pest management (IPM) approaches for pests (Ahmad, 2024), including the house fly, *Musca domestica* Linnaeus, 1758 (Diptera: Muscidae) (Geden et al., 2021). Recognized globally as both a nuisance and a public health pest (Olagunju, 2022; Bertelloni et al., 2023), *M. domestica* has been subjected to control using organophosphates, carbamates, pyrethroids, and newer chemical classes (*e.g.*, phenylpyrazoles, neonicotinoids, insect growth regulators) (Khan, 2021). Resistance to all these classes: organochlorines (Abbasi et al., 2023), organophosphates (Abobakr et al., 2022), carbamates (Khan, 2024), pyrethroids (Scott, 2017), and newer compounds (Shi, Zhang & Gao, 2011) has been detected in *M. domestica* populations worldwide. In Pakistan, resistance and cross-resistance patterns to organophosphates, carbamates, and newer insecticides are particularly evident. Resistance problem has largely attributable to insecticide over-reliance and misuse (Abbas, Khan & Shad, 2014; Khan, Akram & Fatima, 2017; Riaz et al., 2022). As a result, effective management of *M. domestica* has become a growing challenge across the globe, particularly where insecticide performance is compromised.

To effectively manage *M. domestica* populations and combat resistance, it is imperative to elucidate the nature of insecticide resistance, including its mechanisms, inheritance patterns, and cross-resistance profiles (Khan, 2021; Lu, Xu & Wang, 2024). Research into inheritance facilitates resistance monitoring and risk assessment, enabling the implementation of informed resistance management tactics (Tabashnik, 1990). Previous studies have characterized resistance mechanisms, inheritance, and cross-resistance in *M. domestica* strains from various origins (Tian et al., 2011; Li et al., 2013; Khan, Akram & Haider, 2015a; Ma et al., 2017; Freeman & Scott, 2024). However, the principal resistance mechanisms are geographically heterogeneous, influenced by disparate insecticide selection pressures, environmental factors, and strain-specific characteristics (Walsh et al., 2022). This geographic variation is exemplified by deltamethrin resistance: stable without selection in an Indian *Plutella xylostella* (Linnaeus, 1758) strain (Lepidoptera: Plutellidae), yet unstable in strains from Pakistan and Malaysia (Sayyed et al., 2005; Balasubramani, Sayyed & Crickmore, 2008; Sayyed et al., 2008). Moreover, resistance to pyrethroids (cypermethrin, deltamethrin and permethrin) in geographically different Iranian strains of *M. domestica* exhibited multiple resistance phenotypes (Ahmadi, Khajehali & Rameshgar, 2020). For example, according to synergistic analysis, cytochrome P450 monooxygenases (P450s), glutathione S-transferases (GSTs), and carboxylesterases (CarEs) were involved in pyrethroid resistance in the Isfahan strain. In the Mobarake strain, resistance was associated with P450s and GSTs. In the Natanz and Alavijeh strains, CarEs were responsible for detoxifying pyrethroids (Ahmadi, Khajehali & Rameshgar, 2020).

Methomyl, a carbamate insecticide, has been used to control *M. domestica* with reports of resistance development from different countries (Barson, 1989; Chapman & Morgan, 1992; Darbro & Mullens, 2004; Kristensen et al., 2006; White et al., 2007; Memmi, 2010). Although methomyl is banned in various countries due to human health and environmental concerns (Yıldırım & Çiftçi, 2022), it is still used for pest management in several countries, including Pakistan. Recently, field-evolved resistance to methomyl in *M. domestica* has been reported from different localities of Sindh and Punjab, Pakistan (Khan, 2023). Moreover, a laboratory-selected strain of *M. domestica* (Meth-SEL) showed a high risk of resistance development to methomyl along with cross-resistance potential to permethrin. However, there is a lack of information regarding inheritance pattern, or mechanism of resistance in methomyl resistant strains of *M. domestica*. In light of the significant spatio-temporal variation in resistance profiles, the specific objectives of this study were: (1) to develop a near-isogenic line of methomyl-resistant *M. domestica* derived from a Pakistani field strain, and (2) to utilize this line to elucidate the genetic basis, mode of inheritance, and cross-resistance patterns of methomyl resistance. This information is essential for developing effective *M. domestica* and resistance management strategies in Pakistan, with potential applications elsewhere.

## Materials and Methods

### *Musca domestica* strains, chemicals and backcrossing protocol

A Lab-susceptible strain of *M. domestica* was an insecticide susceptible reference strain that was reared without exposure to any chemicals/insecticides for the last 14 years. The Meth-SEL strain was a methomyl-resistant field strain, which was further selected with methomyl in the laboratory for eight consecutive generations (Khan, 2024). The strains were reared following a well-established protocol (Khan, 2021). A near-isogenic line of *M. domestica* resistant to methomyl (Meth-R) was established using the Lab-susceptible and Meth-SEL strains by the backcrossing protocol (Mu et al., 2004; Khan, 2021). The method was performed essentially as described previously (Khan, 2018; Khan, 2021).

Briefly, mass mating was done by introducing 50 pairs of Lab-susceptible females and Meth-SEL males into a mesh cage within six hours of adults’ eclosion. A self-cross between the offspring of Lab-susceptible and Meth-SEL strain yielded offspring designated as the F_1_ progeny. A backcross between F_1_ males with Lab-susceptible females was performed. The offspring of the backcrosses were referred to as the BCnF_1_ progeny (n = number of backcrosses). The self-bred BCnF_1_ progeny yielded BCnF_2_ progeny which were selected with methomyl using the dose level to cause 70% mortality (*i.e.,* LD_70_), and then the surviving males were backcrossed with Lab-susceptible females. The selection bioassays and backcrossing were completed when the LD_50_ values of the self-bred progenies BCnF_2_, BCnF_3_, and BCnF_4_ had become stable. All strains were reared under the laboratory conditions (25 ± 2 °C, 65 ± 5%, 12L: 12D photoperiod) and fed with sugar-milk-based diet as described previously (Khan, 2021).

Technical-grade methomyl, alpha-cypermethrin, fluralaner, pirimiphos-methyl, clothianidin, pipronyl butoxide (PBO) and *S,S,S*-tributyl phosphorotrithioate (DEF) (Chem Service Inc. West Chester, PA, USA) were used in bioassays.

### Selection and bioassays

The protocol of Liu & Yue (2000) was followed in bioassays and selection experiments. The following methodology section has been described as outlined in author’s previous reports (Khan, Akram & Shad, 2014) with a few necessary modifications:

Before starting the selection experiments, a preliminary evaluation of the field collected flies (from the Lodhran city; 29.5467°N, 71.6276°E) was made to determine the lethal dose values for the desired selection process (*i.e.,* 70% mortality). Moreover, these evaluations were made before each subsequent selection for the purpose to maintain 70% mortality selection pressure. For the selection experiment, unmated *M. domestica*, less than a day old, were anesthetized with ether and exposed to methomyl *via* topical application of a drop of insecticide solution in acetone (0.5-microlitre per fly) to the thoracic notum of each fly using a micropipette (0.1–10 µL). Mortality data recorded after 48 h of exposure to insecticides and survivors were used as the parents of the next generation. For bioassays, 5–7 concentrations (causing > 0 and < 100% mortality) of insecticides were prepared as serial dilutions in acetone and replicated three times. The range of concentrations of methomyl in bioassays was as follows: 44.0, 87.5, 175.0, 350.0, 700.0 and 1,400 ng/fly for BC_1_F_1_ and BC_1_F_2_; 87.5, 175, 350, 700, 1,400 and 2,800 ng/fly for BC_2_F_1_ and BC_2_F_2_; 175, 350, 700, 1,400, 2,800 and 5,600 ng/fly for BC_3_F_1_ and BC_3_F_2_; 250, 500, 1,000, 2,000, 4,000 and 8,000 ng/fly for BC_4_F_1_, BC_4_F_2_, BC_4_F_3_ and BC_4_F_4_. The insecticide solutions were applied by topical application to 2–3-day-old female *M. domestica* (*n* = 20 flies per concentration/per replication). The sample size of three replicates × 20 flies per concentration (total 60) was selected following established methodologies in *M. domestica* resistance studies (Khan, Akram & Shad, 2014). Flies in control groups received topical application of acetone only (0.5-microlitre per fly). The treated flies were released in 250 ml plastic jars containing two cotton wicks (two cm) moistened with 20% sugar water solution.

All mortality data were analyzed by Probit analysis (Finney, 1971) using the software PoloPlus to determine median lethal doses (LD_50*s*_) and 95% confidence intervals (CIs). LD_50_ values of the respective bioassays were considered significantly different on the basis of non-overlapping of 95% CIs (Litchfield & Wilcoxon, 1949), and the lethal-dose ratio test (significant when the 95% CI of the ratio excludes 1) (Robertson et al., 2017).

To test the hypothesis that the Meth-R strain has cross-resistance to other insecticides, the Meth-SEL strain, before starting backcross experiments for the construction of near-isogenic line, was bioassayed with alpha-cypermethrin, fluralaner, pirimiphos-methyl, and clothianidin, and their LD_50_ values calculated. After completion of the backcross experiments, the Meth-R strain was bioassayed again with these insecticides, and LD_50_ values were compared with those obtained for the Meth-SEL strain. LD_50_ values were considered significantly different on the basis of non-overlapping of 95% CIs, and the lethal-dose ratio test (significant when the 95% CI of the ratio excludes 1). Cross-resistance was assumed if the Meth-R strain showed significantly reduced toxicity to other insecticides compared with the Meth-SEL strain.

### Genetic crosses

The genetic crosses were performed as outlined in author’s previous report (Khan, Akram & Shad, 2014):

For the purpose to study the mode of inheritance to methomyl in *M. domestica*, the male and female *M. domestica* were separated immediately after adult eclosion. Reciprocal crosses between the Lab-susceptible and Meth-R strains were made to produce two progenies: F_1_ (Lab-susceptible♀ × Meth-R♂) and *F*_1′_ (Meth-R ♀ × Lab-susceptible ♂). These two progenies were crossed with parental strains to produce four backcross strains: BC_1_ (F_1_♀ × Lab-susceptible♂), BC_2_ (F_1_♀ × Meth-R♂), BC_3_ (*F*_1′_♀ × Lab-susceptible♂), and BC_4_(*F*_1′_♀ × Meth-R♂). In addition, self-bred F_2_ (F_1_♀ × F_1_♂) and F_2_’ (F_1_’♀ × F_1_’♂) progenies were also produced (Khan, Akram & Shad, 2014). The range of concentrations in bioassays with backcross and self-bred progenies were as follows: 62.5, 125, 250, 500, 1,000 and 2,000 ng/fly for BC_1_ and BC_3_; 125, 500, 1,000, 2,000 and 4,000 ng/fly for BC_2_ and BC_4_; 250 to 8,000 ng/fly for F_2_ and *F*_2′_.

### Inheritance pattern analyses

Inheritance pattern analyses were performed as outlined in previous report (Khan, Akram & Shad, 2014):

Firstly, by following the methodology of Sokal & Rohlf (1981). A null hypothesis of monogenic resistance was tested by analyzing the responses of backcross and self-bred progenies to methomyl. The model was evaluated on the basis of chi-square goodness of fit test between expected and observed mortalities as follows:

\begin{eqnarray*}{\alpha }^{2}=({N}_{i}-p{n}_{i})^{2}/pq{n}_{i} \end{eqnarray*}

where *N*_*i*_ and *n*_*i*_ are the observed mortality, and the number of flies exposed to a given dose, respectively. *p* is the expected mortality and *q* = 1 − *p*, as described by Georghiou (1969). The hypothesis was then tested by calculating the sum of Chi-square as${\chi }^{2}=\sum {\mathrm{N}}_{\mathrm{i}}{\alpha }_{\mathrm{i}}^{2}$, and comparing the sum with tabular values from a chi-square table with *N* degrees of freedom. The hypothesis would be accepted if there was no significant difference between observed and expected mortalities (Sokal & Rohlf, 1981; Shi, Zhang & Gao, 2011).

The second approach was to compare log dose-probit lines of the strains from backcross and self-bred crosses. If the log dose-probit lines of the susceptible strain, resistant strain and their reciprocal strains do not overlap, the hypothesis of monogenic resistance will be accepted if plateaus in the log dose probit lines of the self-bred occur at about 25% (probit value 4.33) and 75% mortality (probit value 5.67), and in the backcross strains at about 50% mortality (probit value 5) (Tsukamoto, 1983). For this, log dose-probit lines were plotted by taking log of concentrations and converting percent mortalities to probit values (Khan, 2021).

### Synergism bioassays

In synergism bioassays, the enzyme inhibitors PBO and DEF were topically applied to *M. domestica* at a maximum sublethal dose of 10 µg per fly (Liu & Yue, 2000; Khan, Akram & Iqbal, 2015b). One hour later, flies were treated with methomyl doses. The synergism ratio (SR) was calculated by dividing the LD_50_ of methomyl alone for a strain by the LD_50_ of methomyl plus PBO or DEF for the same strain. An SR was considered significantly different if its 95% CI value did not include one, based on the ratio test (Robertson et al., 2017).

## Results

### Development of Meth-R near-isogenic line of *Musca domestica*

Prior to backcrossing experiments, the Lab-susceptible and Meth-SEL strains of *M. domestica* exhibited LD_50_ values of methomyl as 0.56 and 2,116.70 ng/fly, respectively (Table 1). The Meth-SEL strain was 3,779.82-fold resistant to methomyl when compared with the Lab-susceptible strain. The results of bioassays of backcross progenies revealed that the LD_50_ and RR values were significantly lower in the F_1_ stage of BC_1_ and BC_2_, when the backcross was performed with the recurrent susceptible strain, than those observed in the self-bred progenies (*F*_2s_). For example, the LD_50_ values increased from 285.14 to 641.17, and 1,060.80 to 2,142.50 ng/fly in BC_1_ and BC_2_, respectively. There was no significant difference among the LD_50_ values of BC_3-4_F_1_ and subsequent progenies. The stable LD_50_ values indicated the development of methomyl-resistant (Meth-R) near-isogenic line of *M. domestica* (Table 1).

**Table 1:** Median lethal doses (LD_50s_) with 95% confidence interval for methomyl in parent and backcross progenies during the construction of the near-isogenic line of *Musca domestica* resistant to methomyl.

**Strain**	**LD**_50_ ** (95% CI)(ng/fly)**	**Fit of probit line**				RR^**^
		**Slope (SE)**	*χ* ^2^	df^£^	***P***	
Lab-susceptible	0.56 (0.40–0.80)	2.27 (0.23)	3.95	4	0.41	
Meth-SEL^*^	2,116.70 (1,717.04–2,732.82)	1.87 (0.19)	1.79	4	0.77	3,779.82 (2,830.43–5,036.14)
F_1_	311.93 (216.75–534.27)	1.92 (0.20)	6.63	4	0.16	557.02 (417.57–741.35)
BC_1_F_1_	285.14 (237.88–343.42)	2.00 (0.17)	3.24	4	0.52	509.18 (395.11–654.68)
BC_1_F_2_	641.17 (516.15–833.03)	1.72 (0.18)	1.94	4	0.75	1,144.94 (852.47–1534.26)
BC_2_F_1_	1,060.80 (887.37–1294.50)	2.27 (0.23)	3.95	4	0.41	1,894.29 (1,465.00–2,443.75)
BC_2_F_2_	2,142.50 (1,651.49–3,110.19)	1.71 (0.33)	1.73	3	0.63	3,822.32 (2,689.11–5,430.82)
BC_3_F_1_	4,349.38 (3,163.56–6,871.47)	1.35 (0.18)	0.18	4	0.99	7,766.75 (5,126.52–11,739.84)
BC_3_F_2_	8,558.90 (5,417.00–19,954.00)	1.31 (0.23)	1.52	3	0.68	15,283.75 (8,211.03–28,383.58)
BC_4_F_1_	8,684.50 (6,004.90–15,397.00)	1.35 (0.19)	0.30	4	0.99	15,508.04 (9,557.76–25,104.89)
BC_4_F_2_	8,920.30 (6,193.00–15,708.00)	1.40 (0.25)	1.02	4	0.91	15,929.11 (9,863.53–25,665.64)
BC_4_F_3_	8,864.21 (6,011.41–16,303.01)	1.28 (0.18)	0.39	4	0.98	15,828.95 (9,522.62–26,251.55)
BC_4_F_4_ (Meth-R)	8,686.80 (6,320.01–13,741.00)	1.35 (0.19)	0.06	4	0.99	15,512.14 (9,552.91–25,130.97)

**Notes.**

* Reported previously (Khan, 2024).

** Resistance ratio.

£ Number of insects used in each bioassay (including control) were 360 (for *df* = 3) or 420 (for *df* = 4).

### Inheritance analyses of methomyl resistance

**Figure 1:**
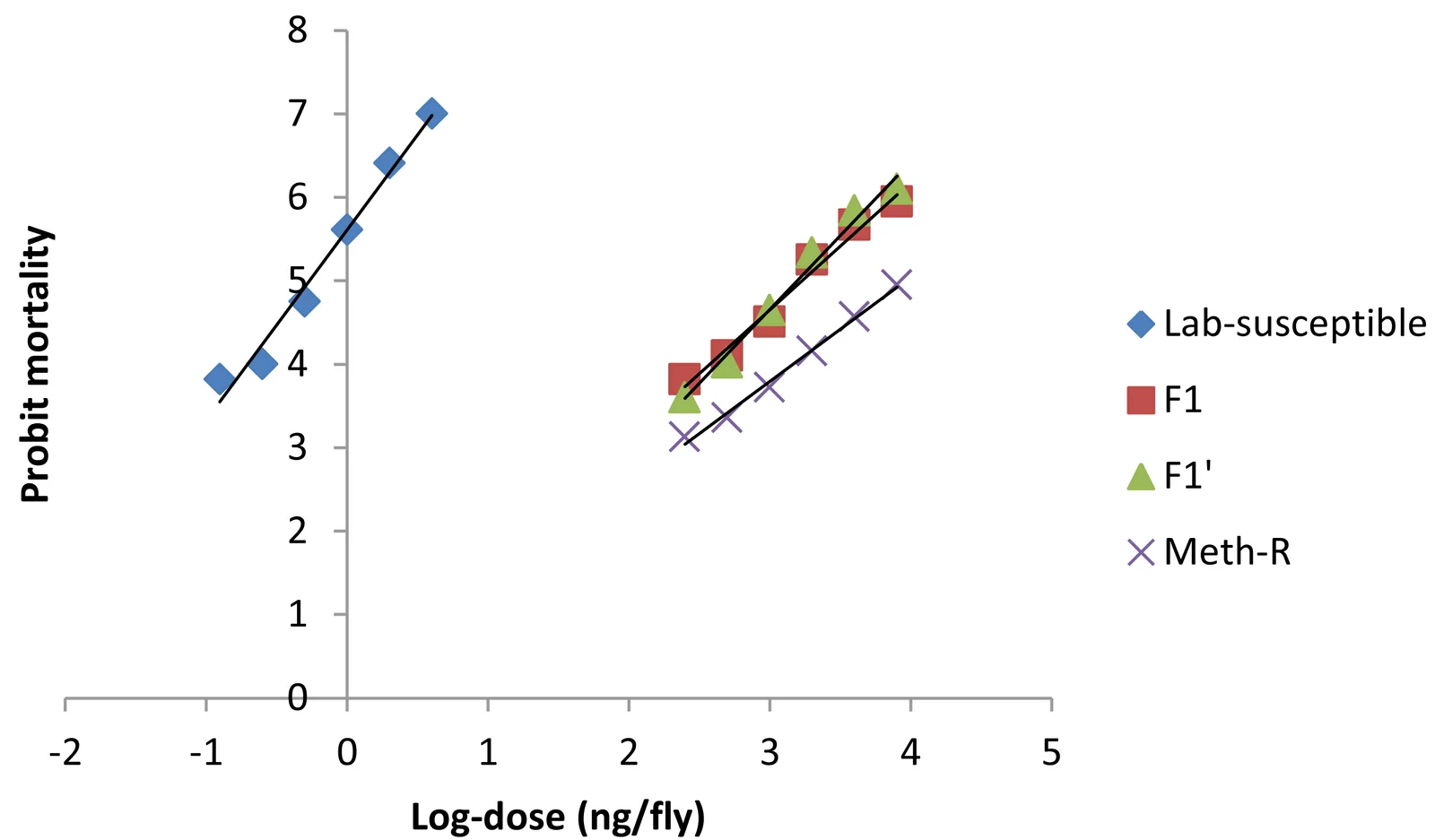
Log-dose probit lines for Lab-susceptible, Meth-R, and their reciprocal (F1, and F1′) progenies of *Musca domestica* after exposure to different doses of methomyl. Number of insects used in each bioassay for each progeny were 420. Slope ± (SE) values were 2.27 (0.23) for Lab-susceptible; 1.35 (0.19) for Meth-R; 1.57 (0.16) for F1; 1.79 (0.17) for F1′.

Based on the straight shapes of log-dose probit lines of Lab-susceptible, Meth-R, and F_1_ and *F*_1′_ (reciprocal progenies) of *M. domestica* (Fig. 1) and non-significant *χ*^2^ values (Table 2), it is evident that both the Lab-susceptible and Meth-R strains of *M. domestica* were homogeneously susceptible and resistant to methomyl, respectively. The LD_50_ values of methomyl for the self-bred progenies F_1_ (Lab-susceptible♀ × Meth-R♂) and *F*_1′_ (Meth-R♀ × Lab-susceptible♂) were recorded to be 1,667.28 and 1,577.30 ng/fly, respectively, which were statistically at par (Table 2). Since reciprocal crosses [(Lab-susceptible♀ × Meth-R♂) and (Meth-R♀ × Lab-susceptible♂)] yielding similar LD_5__0_s rule out both sex-linked inheritance (which would differ between male and female progeny) and maternal/cytoplasmic effects (which would elevate resistance in progeny derived from resistant females), these data collectively indicate that methomyl resistance in the Meth-R strain is autosomal. Moreover, the degree of dominance (D) value for the F_1_ and *F*_1′_ progenies were 0.70 and 0.60, respectively, suggesting that resistance to methomyl was inherited as an incompletely dominant trait in the Meth-R strain of *M. domestica* (Table 2).

**Figure 2:**
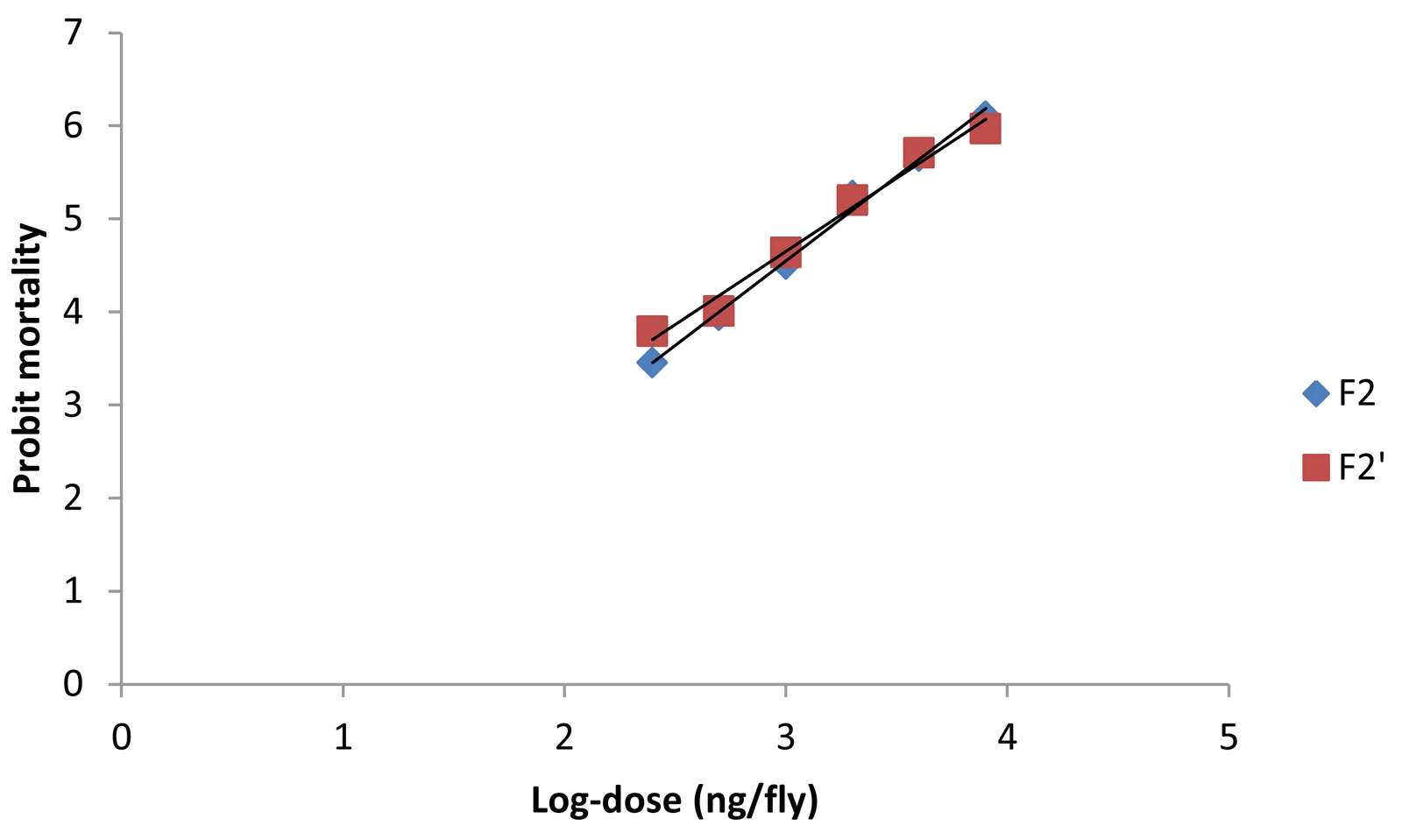
Log-dose probit line for the self-bred (F2 and F2′) progenies of *Musca domestica* after exposure to different doses of methomyl. Number of insects used in each bioassay for each progeny were 480. Slope ± (SE) values were 1.81 (0.16) for F2 ; 1.58 (0.15) for F2′.

**Figure 3:**
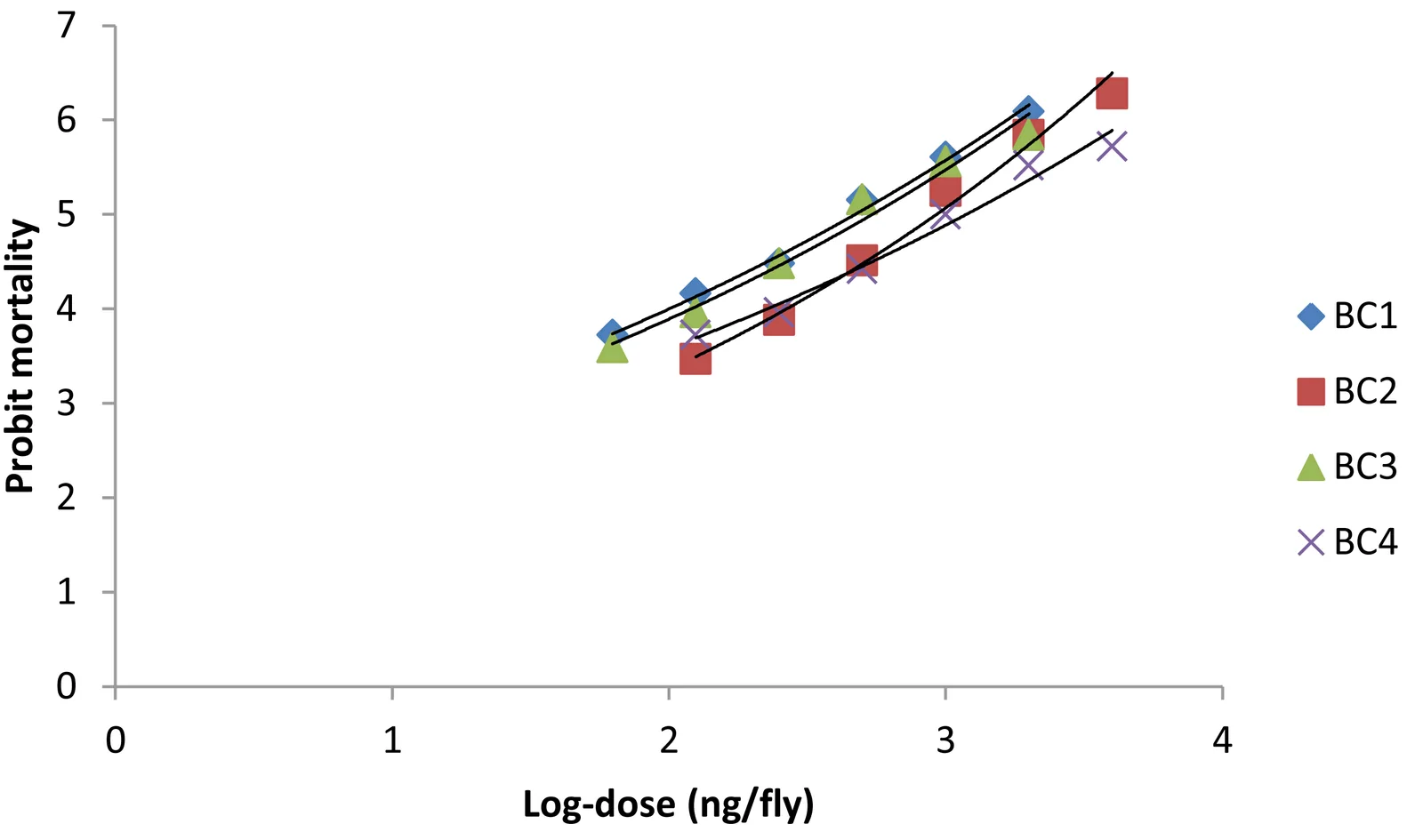
Log-dose probit lines of backcrosses (BC1, BC2, BC3, and BC4) after exposure to different doses of methomyl. Number of insects used in each bioassay for each progeny were 480. Slope ± (SE) values were 1.63 (0.23) for BC1; 1.98 (0.18) for BC2; 1.59 (0.16) for BC3; 1.47 (0.15) for BC4.

Toxicity responses of self-bred F_2_ (F_1_♀ × F_1_♂) and *F*_2′_ (*F*_1′_♀ × *F*_1′_♂) progenies, and four backcross BC_1_, BC_2_, BC_3_ and BC_4_ strains to methomyl were calculated as 1,721.20, 1,624.07, 425.16, 811.36, 485.54 and 1,083.58 ng/fly, respectively (Table 3). The offspring of F_2_ and *F*_2′_, and BC_1−4_ strains showed significant deviation from the monofactorial model when assayed with methomyl (*χ*^2^*df* = 5 = 219.43, 240.85, 432.33, 358.21, 402.48 and 256.72, respectively; *p* < 0.05 in all cases). These data suggest that methomyl resistance in the Meth-R strain was controlled by more than one factor. In addition, the absence of plateaus in the log-dose Probit lines of all these progenies (Figs. 1–3) further indicated that methomyl resistance in the Meth-R strain was controlled by more than one factor.

**Table 2:** Median lethal doses (LD_50s_) with 95% confidence interval for methomyl in Lab- susceptible, Meth-R strains and their reciprocal progenies of *Musca domestica*.

**Strain**	**LD**_**50**_ **(95% CI) (ng/fly)**	**Slope (SE)**	*χ* ^2^	**df** ^£^	***P***	RR^*^	Dominance value
Lab-susceptible	0.56 (0.40–0.80)	2.27 (0.23)	3.95	4	0.41		
Meth-R	8,686.80 (6,320.01–13,741.00)	1.35 (0.19)	0.06	4	0.99	15,512.14	
F_1_ (Lab-susceptible♀× Meth-R♂)	1,667.28 (1,346.08–2,080.91)	1.57 (0.16)	2.10	4	0.72	2,977.29	0.70
F_1′_ (Meth-R♀× Lab-susceptible♂)	1,577.30 (1,248.88–1,846.40)	1.79 (0.17)	1.95	4	0.74	2,816.61	0.60

**Notes.**

£ Number of insects used in each bioassay were 420.

* Resistance ratio.

**Table 3:** Median lethal doses (LD_50s_) with 95% confidence intervals for methomyl and chi-square analysis for monogenic inheritance of resistance in self-bred and backcross progenies of *Musca domestica*.

Strain^£^	LD_50_ (95% CI) (ng/fly)	Slope (SE)	RR^*^	${\chi }^{2}={\mathop{\sum }\nolimits }_{df=5}^{{N}_{i}{\alpha }_{i}^{2}}$
F_2_ (F1♀× F1♂)	1,721.20 (1,421.19–2,097.04)	1.81 (0.16)	3,073.57	219.43
*F*_2′_ (F1′♀× F1′♂)	1,624.07 (1,312.45-2,021.85)	1.58 (0.15)	2,900.13	240.85
BC_1_ (F1♀× Lab-susceptible♂)	425.16 (345.68–527.30)	1.63 (0.23)	759.21	432.33
BC_2_ (F1♀× Meth-R♂)	811.36 (677.89–974.41)	1.98 (0.18)	1,448.86	358.21
BC_3_(F1′♀× Lab-susceptible♂)	485.54 (393.18–608.02)	1.59 (0.16)	867.04	402.48
BC_4_ (F1′♀× Meth-R♂)	1,083.58 (864.62–1,390.76)	1.47 (0.15)	1,934.96	256.72

**Notes.**

£ Number of insects used in each bioassay including control were 480.

* Resistance ratio = LD_50_ of F_2_, *F*_2′_ or BC_1, 2, 3, or 4_/LD_50_ of Lab-susceptible.

### Cross-resistance analysis

The LD_50_ values of alpha-cypermethrin, fluralaner, pirimiphos-methyl, and clothianidin prior to backcrossing experiments against the Meth-SEL strain were non-significantly different from those recorded against the Meth-R strain (Table 4). Although there was cross-resistance in the Meth-SEL strain to alpha-cypermethrin, fluralaner, pirimiphos-methyl, and clothianidin *versus* the lab susceptible strain, selection with methomyl did not increase the level of cross-resistance.

**Table 4:** Bioassays for cross-resistance of Meth-R strain of *Musca domestica* to different insecticides. Data are median lethal doses (LD_50s_) with 95% confidence intervals.

**Insecticide**	**Strain** ^£^	**LD**_**50**_ ** (95% CI) (ng/fly)**	**Slope (SE)**	RR^*^
Alpha-cypermethrin	Lab-susceptible	2.51 (1.71–3.88)	2.34 (0.23)	1
	Meth-SEL	70.47 (59.57–84.17)	2.21 (0.20)	28.08
	Meth-R	69.53 (57.49–87.89)	1.84 (0.17)	27.70
Fluralaner	Lab-susceptible	1.26 (0.90–1.83)	2.31 (0.22)	1
	Meth-SEL	8.00 (4.93–13.19)	2.28 (0.23)	6.35
	Meth-R	9.24 (6.51–13.42)	2.39 (0.27)	7.33
Pirimiphos-methyl	Lab-susceptible	1.02 (0.71–1.47)	2.01 (0.21)	1
	Meth-SEL	9.07 (6.48–13.03)	2.31 (0.23)	8.89
	Meth-R	10.40 (6.57–18.67)	2.29 (0.31)	10.19
Clothianidin	Lab-susceptible	0.78 (0.57–1.13)	2.40 (0.24)	1
	Meth-SEL	6.79 (5.40–8.98)	1.56 (0.17)	8.71
	Meth-R	7.27 (5.55–10.27)	1.32 (0.16)	9.32

**Notes.**

£ Number of insects used in each bioassay (including control) were 360 (for pirimiphos methyl and clothianidin) or 420 (for alpha-cypermethrin and fluralaner).

* Resistance ratio.

### Synergism of PBO and DEF

Bioassays of methomyl in combination with PBO or DEF did not cause significant change in the LD_50_ values of the Lab-susceptible strain on the bases of non-significant change in synergism ratios (SRs) (Table 5). However, in the cases of Meth-SEL and Meth-R strains, toxicity of methomyl significantly increased in the presence of PBO or DEF. The toxicity of methomyl alone in the Meth-SEL strain was 2,116.70 ng/fly, compared to 923.19 ng/fly for methomyl+PBO and 593.13 ng/fly for methomyl+DEF, yielding SRs of 2.29 and 3.57, respectively. Similarly, the toxicity of methomyl alone in the Meth-R strain was 8,686.80 ng/fly, compared to 1,719.06 ng/fly for methomyl+PBO and 1,693.49 ng/fly for methomyl+DEF, resulting in SRs of 5.05 and 5.13, respectively.

**Table 5:** Synergistic effect of PBO and DEF on the m edian lethal doses (LD_50_) with 95% confidence intervals for methomyl in different *Musca domestica* strains.

**Compound**	**Strain**	**LD**_**50**_**(95% CI)** (ng/fly)	**Fit of probit line**				SR^*^
			**Slope (SE)**	*χ* ^2^	**df** ^£^	***P***	
Methomyl	Lab-susceptible	0.56 (0.40–0.80)	2.27 (0.23)	3.95	4	0.41	–
Methomyl+PBO		0.64 (0.40–1.14)	1.83 (0.20)	5.69	4	0.22	0.88 (0.66–1.12)
Methomyl+DEF		0.70 (0.48–1.14)	1.53 (0.19)	3.06	4	0.55	0.80 (0.59–1.06)
Methomyl	Meth-SEL	2,116.70 (1,717.04–2,732.82)	1.87 (0.19)	1.79	4	0.77	–
Methomyl+PBO		923.19 (737.93–1,214.99)	1.66 (0.21)	0.87	4	0.93	2.29 (1.64–3.21)
Methomyl+DEF		593.13 (488.98–725.56)	1.91 (0.21)	1.86	4	0.76	3.57 (2.64–4.83)
Methomyl	Meth-R	8,686.80 (6,320.01–13,741.00)	1.35 (0.19)	0.06	4	0.99	–
Methomyl+PBO		1,719.06 (1,431.37–2,118.46)	2.11 (0.23)	1.92	4	0.75	5.05 (3.09–8.27)
Methomyl+DEF		1,693.49 (1,347.13–2,233.73)	1.59 (0.20)	0.85	4	0.93	5.13 (3.06–8.59)

**Notes.**

* LD_50_ of methomyl alone/LD_50_ of methomyl with PBO or DEF.

£ Number of insects used in each bioassay were 420.

## Discussion

It is imperative to generate an insecticide resistant near-isogenic line of a particular insect pest in order to study inheritance pattern, cross-resistance analysis and mechanism of insecticide resistance (Khan, 2021). A near-isogenic line would have a similar or near to similar genetic background of its recurrent parental strain with a minor difference in limited genes, including the genes of interest (*i.e.,* methomyl resistance in the present case). In this paper, the Meth-R strain was generated by the backcrossing inbred line protocol using the Meth-SEL and Lab-susceptible strains of *M. domestica*. The Meth-SEL and Lab-susceptible strains were ideally suitable parents to generate the near-isogenic line because of their homogenous resistant and susceptible nature, respectively, which have been demonstrated by the shape of their log-dose probit lines. The development of the methomyl resistant near-isogenic line was involved crossing, backcrossing, and self-breeding (Shan et al., 2016). During crossing technique, the exchange of genetic material between recurrent and non-recurrent parent is expected to happen. Repeated backcrossing of the hybrids with the recurrent parental strain and subsequent selection with methomyl results in the elimination of genes from the non-recurrent parental strain. The self-breeding helps to integrate resistant-target genes into recurrent parent’s genetic make-up that ultimately persist in the resultant near-isogenic line (Zhu et al., 2015; Khan, 2021). Hence, insecticide resistant isogenic lines, Meth-R in the present study, would be the best fit to study mode of inheritance and further characterize resistance to methomyl owing to its similar genetic makeup as that of its recurrent parent, except for the resistant target gene(s).

The ecological significance of methomyl resistance in *M. domestica* should be viewed against its widespread use in Pakistani agriculture, particularly in cotton, where *M. domestica* occur as a non-target species. Field strains from cotton-growing areas of Punjab and Sindh have shown methomyl resistance ratios of 28- to 137-fold relative to susceptible laboratory strains, with LD_50_ values as high as 136 µg fly^−1^. The Meth-R strain’s LD_50_ (8,686.8 ng/fly) exceeds this reported range. Considering that recommended application rates in Pakistan are 300–741 g a.i. ha^−1^ and that residues on treated surfaces typically range from 1.0 to 15 µg g^−1^ (El-Hefny et al., 2019), the resistance level we observed would probably compromise methomyl efficacy in the field, especially given the insecticide’s rapid dissipation (half-life < 2 days in soil) (El-Hefny et al., 2019). These comparisons confirm that the resistance documented here is not merely a laboratory phenomenon but has important implications for methomyl-based control strategies.

Study of the mode of inheritance of insecticide resistance is of practical significance in deciding how effectively an insecticide can be used to manage resistant insect pests (Bouvier et al., 2001). Inheritance pattern of insecticide resistance such as dominant or recessive, monogenic or polygenic, sex-linked or autosomal can be traced by studying toxicity responses of the progenies resulted after the reciprocal crosses between male and females of susceptible and resistant strains (Lu, Xu & Wang, 2024). The results of the present study exhibited that the LD_50_ values and log-dose probit line of the progenies of reciprocal crosses between male and females of susceptible and resistant strains (F_1_ and *F*_1′_) were non-significantly different, which provided clues that methomyl resistance in the Meth-R strain was autosomal and without maternal or sex linkage effects. In addition, degree of dominance values of these reciprocal crosses suggested that methomyl resistance was inherited in incompletely dominant pattern. The dominance values (*D* = 0.70, 0.60) were calculated as point estimates using Stone (1968) formula. This deterministic method does not generate confidence intervals, and most inheritance studies report D values without them (Abbas, Khan & Shad, 2014; Khan, 2021). Nonetheless, the incomplete dominance interpretation is reinforced by the intermediate F_1_ and F_1_′ dose–response curves relative to the parental strains. Furthermore, resistance to methomyl in the Meth-R strain of *M. domestica* was polyfactorial. Results of the present study are in broad agreement with the inheritance patterns of carbamates in other insects. For example, resistance to carbamate in *Aphytis melinus* DeBach, 1959 (the California red scale parasite) was polyfactorial with recessive mode of inheritance (Spollen & Hoy, 1992). However, in contrast to the results of present study, sex-linkage effects of resistance to carbaryl have been identified in *A. melinus* (Spollen & Hoy, 1992). Moreover, resistance to carbamate was inherited as a dominant trait in different insect pests (Mougabure-Cueto & Picollo, 2015; Onstad & Gassmann, 2023). Resistance to another carbamate insecticide (propoxur) in a near-isogenic line of *M. domestica* was inherited as a monofactorial, autosomal, and an incompletely recessive trait (Shan et al., 2016). Resistance inheritance of propoxur in *Anopheles stephensi* Liston, 1901, was reported as autosomal, monofactorial and incompletely dominant trait. Monofactorial resistance to propoxur was also reported in *Blattella germanica* Linnaeus, 1767 (Katz, Collins & Skavaril, 1973). It has been reported that the inheritance patterns or nature of resistance to a particular insecticide class or insecticides within the same class could be variable depending upon insecticide exposure histories, origin of species with a specific genetic makeup, and selection bioassays under laboratory conditions (Khan, Akram & Haider, 2015a; Shan et al., 2016; Khan, 2020; Lu, Xu & Wang, 2024).

The data of the present study revealed incomplete dominant inheritance of methomyl in the Meth-R strain of *M. domestica*. From the management perspectives, the presence of dominant mode of inheritance of insecticide resistance is more problematic than that of recessive pattern of inheritance since heterozygotes would not be easily controlled under field conditions (Zhang et al., 2021). Control of heterozygous resistant insects with dominant mode of inheritance is usually required high dosage of insecticides than the label recommendations, in order to avoid conversion of incomplete dominance to complete dominance (Roush, 1989; Bourguet, Genissel & Raymond, 2000). Studies revealed that resistance to insecticides with dominant inheritance has the tendency to spread rapidly even if resistance to a particular insecticide is very low in the field (Roush & McKenzie, 1987; Sayyed & Wright, 2006). Hence, keeping in view mode of inheritance in the present study, responsible use of methomyl is needed to lessen severity of the resistance problem.

Besides mode of inheritance, determining number of genes involved in the development of resistance play a critical role in resistance management (Hobbs, Weetman & Hastings, 2023). The data of the present study exhibited that resistance to methomyl in the Meth-R strain of *M. domestica* was probably influenced by more than one gene. Theoretically speaking, it is believed that insecticide resistance with monogenic inheritance pattern spread faster than polygenic; however, in both scenarios, management of resistance requires a lot of efforts and cost (Schechtman & Souza, 2015; Khan, 2021; Lu, Xu & Wang, 2024). Various field strain of *M. domestica* have recently been reported to develop moderate to high levels of field-evolved resistance to methomyl (Khan, 2023), which requires attention for the effective management of resistance along with *M. domestica*.

Assessing the possibility of cross-resistance in resistant strains to other insecticide could be helpful in selecting appropriate candidates for the management of resistant insect species (Liu & Yue, 2000). In the present work, the Meth-R strain did not show cross-resistance to alpha-cypermethrin, fluralaner, pirimiphos-methyl, and clothianidin. So far, there have been variable reports on cross-resistance of methomyl resistant insects to other conventional insecticides. For example, a Korean strain of methomyl-resistant *M. domestica* (Met-R16 strain) showed cross-resistance to several insecticides in carbamate, organophosphate and pyrethroid classes (Yoo et al., 2001). Dağlıand Tunç (2008) found a low level of cross-resistance between cypermethrin and methomyl in *Frankliniella occidentalis* Pergande, 1895. Recently, a methomyl resistant strain of *M. domestica* (Meth-SEL) showed cross-resistance to permethrin; however, this strain did not show cross-resistance to profenofos, imidacloprid and spinetoram (Khan, 2024). Several researchers reported that insect pests usually develop cross-resistance to insecticides with different mode of action under field conditions because of exposure to mixed insecticides in field (Shono, Zhang & Scott, 2004; Pu et al., 2010; Scott, Tolman & MacArthur, 2015; Sang et al., 2016; Qian et al., 2024; Qu et al., 2024). Since the Meth-R strain of *M. domestica* did not show cross-resistance to different insecticides, it provides an opportunity to consider the possible use of these insecticides as alternatives for the management of methomyl-resistant *M. domestica*. However, further research should be conducted in this regard before making any recommendations.

Enhanced metabolism in resistant-insect species is one of well-known and important mechanisms responsible to confer resistance to insecticides (Siddiqui et al., 2023; Pu & Chung, 2024; Liang et al., 2025). Recently, Khan (2023) reported significant synergism of methomyl toxicity by the enzyme inhibitors PBO and DEF in several methomyl-resistant field strains of *M. domestica* from Punjab and Sindh provinces of Pakistan, which provided clues of metabolic mechanisms of resistance. The results of the present study also exhibited that PBO or DEF significantly synergized toxicity of methomyl in Meth-SEL and Meth-R strains of *M. domestica*. Thus, a metabolic mechanism may play a role in methomyl resistance in the studied strains of *M. domestica*. Further *in vitro* experiments could be helpful to confirm the involvement of enhanced metabolism responsible for methomyl resistance in *M. domestica*.

## Limitations

A limitation of this study is that for the BC_4_Fn and Meth-R strains, the LD_50_ values for methomyl exceeded the highest tested doses, requiring extrapolation. This was due to the unexpectedly high resistance level in these strains. Future studies should use wider dose ranges to obtain interpolated LD_50_ values. Additionally, although probit analysis is the standard method in this field, we acknowledge that it does not account for replicate-level random variation. Future studies would benefit from applying generalized linear mixed-effects models (GLMM) to provide more robust estimates of resistance parameters.

## Conclusion

In conclusion, resistance to methomyl in *M. domestica* was inherited as autosomal, polyfactorial, and incompletely dominant trait. The resistant strains of *M. domestica* did not appear to show cross-resistance to insecticides tested in the present experiments. Moreover, synergized toxicity of methomyl by the enzyme inhibitors PBO and DEF revealed the probable presence of metabolic mechanism of resistance. Keeping in view moderate to high levels of resistance to methomyl in *M. domestica* from Pakistan, the cautious use of alternative chemicals along with integrated measures for the management of *M. domestica* are needed. The findings of the present study could be helpful for designing the resistance management strategy and preventing severity of methomyl resistance-crisis in the future.

## Supplemental Information

10.7717/peerj.21646/supp-1 Supplemental Information 1 Mortality data in different treatments

## Additional Information and Declarations

### Data Availability

The following information was supplied regarding data availability: The raw measurements are available in the Supplemental File.

## References

[ref-1] Abbas N, Khan HAA, Shad SA (2014). Resistance of the house fly *Musca domestica* (Diptera: Muscidae) to lambda-cyhalothrin: mode of inheritance, realized heritability, and cross-resistance to other insecticides. Ecotoxicology.

[ref-2] Abbasi E, Yazdani Z, Daliri S, Moemenbellah-Fard MD (2023). Organochlorine knockdown-resistance (kdr) association in housefly (*Musca domestica*): a systematic review and meta-analysis. Parasite Epidemiology and Control.

[ref-3] Abobakr Y, Al-Hussein FI, Bayoumi AE, Alzabib AA, Al-Sarar AS (2022). Organophosphate Insecticides resistance in field populations of house flies, *Musca domestica* L.: levels of resistance and acetylcholinesterase activity. Insects.

[ref-4] Ahmad N (2024). Pest management in agriculture: integrated approaches for sustainable control. Frontiers in Agriculture.

[ref-5] Ahmadi E, Khajehali J, Rameshgar F (2020). Evaluation of resistance to permethrin, cypermethrin and deltamethrin in different populations of *Musca domestica* (L.), collected from the Iranian dairy cattle farms. Journal of Asia-Pacific Entomology.

[ref-6] Balasubramani V, Sayyed AH, Crickmore N (2008). Genetic characterization of resistance to deltamethrin in *Plutella xylostella* (Lepidoptera: Plutellidae) from India. Journal of Economic Entomology.

[ref-7] Barson G (1989). Response of insecticide-resistant and susceptible houseflies (*Musca domestica*) to a commercial granular bait formulation containing methomyl. Medical and Veterinary Entomology.

[ref-8] Bertelloni F, Bresciani F, Cagnoli G, Scotti B, Lazzerini L, Marcucci M, Colombani G, Bilei S, Bossù T, De Marchis ML (2023). House flies (*Musca domestica*) from swine and poultry farms carrying antimicrobial resistant Enterobacteriaceae and Salmonella. Veterinary Sciences.

[ref-9] Bourguet D, Genissel A, Raymond M (2000). Insecticide resistance and dominance levels. Journal of Economic Entomology.

[ref-10] Bouvier J-C, Buès R, Boivin T, Boudinhon L, Beslay D, Sauphanor B (2001). Deltamethrin resistance in the codling moth (Lepidoptera: Tortricidae): inheritance and number of genes involved. Heredity.

[ref-11] Chapman PA, Morgan CP (1992). Insecticide resistance in *Musca domestica* L. from eastern England. Pesticide Science.

[ref-12] Darbro JM, Mullens BA (2004). Assessing insecticide resistance and aversion to methomyl-treated toxic baits in *Musca domestica* L (Diptera: Muscidae) populations in southern California. Pest Management Science.

[ref-13] Dağlı F, Tunç I (2008). Insecticide resistance in *Frankliniella occidentalis*: Corroboration of laboratory assays with field data and cross-resistance in a cypermethrin-resistant strain. Phytoparasitica.

[ref-14] El-Hefny D, Abdallah I, Helmy R, Mahmoud H (2019). Dissipation of methomyl residues in tomato fruits, soil and water using LC-MS/MS. Journal of Plant Protection Research.

[ref-15] Finney D (1971). A statistical treatment of the sigmoid response curve. Probit analysis.

[ref-16] Freeman JC, Scott JG (2024). Genetics, genomics and mechanisms responsible for high levels of pyrethroid resistance in *Musca domestica*. Pesticide Biochemistry and Physiology.

[ref-17] Geden C, Nayduch D, Scott J, Burgess IV E, Gerry A, Kaufman P, Thomson J, Pickens V, Machtinger E (2021). House fly (Diptera: Muscidae): biology, pest status, current management prospects, and research needs. Journal of Integrated Pest Management.

[ref-18] Georghiou G (1969). Genetics of resistance to insecticides in houseflies and mosquitoes. Experimental Parasitology.

[ref-19] Hobbs NP, Weetman D, Hastings IM (2023). Insecticide resistance management strategies for public health control of mosquitoes exhibiting polygenic resistance: A comparison of sequences, rotations, and mixtures. Evolutionary Applications.

[ref-20] Katz A, Collins W, Skavaril R (1973). Resistance in the German cockroach (Orthoptera: Blattellidae): the inheritance of diazinon resistance and cross resistance. Journal of Medical Entomology.

[ref-21] Khan HAA (2018). Spinosad resistance affects biological parameters of *Musca domestica* Linnaeus. Scientific Reports.

[ref-22] Khan HAA (2020). Susceptibility to indoxacarb and synergism by enzyme inhibitors in laboratory and field strains of five major stored product insects in Pakistan. Chemosphere.

[ref-23] Khan HAA (2021). Permethrin resistance associated with inherited genes in a near-isogenic line of *Musca domestica*. Pest Management Science.

[ref-24] Khan HAA (2023). Monitoring resistance to methomyl and synergism in the non-target *Musca domestica* from cotton fields of Punjab and Sindh provinces, Pakistan. Scientific Reports.

[ref-25] Khan HAA (2024). Resistance risk assessment, cross-resistance potential and realized heritability of resistance to methomyl in *Musca domestica* Linnaeus. Ecotoxicology.

[ref-26] Khan HAA, Akram W, Fatima A (2017). Resistance to pyrethroid insecticides in house flies, *Musca domestica* L. (Diptera: Muscidae) collected from urban areas in Punjab, Pakistan. Parasitology Research.

[ref-27] Khan HAA, Akram W, Haider MS (2015a). Genetics and mechanism of resistance to deltamethrin in the house fly, *Musca domestica* L. from Pakistan. Ecotoxicology.

[ref-28] Khan HAA, Akram W, Iqbal N (2015b). Selection and preliminary mechanism of resistance to profenofos in a field strain of *Musca domestica* (Diptera: Muscidae) from Pakistan. Journal of Medical Entomology.

[ref-29] Khan HA, Akram W, Shad SA (2014). Genetics, cross-resistance and mechanism of resistance to spinosad in a field strain of *Musca domestica* L. (Diptera: Muscidae). Acta Tropica.

[ref-30] Kristensen M, Huang J, Qiao CL, Jespersen JB (2006). Variation of *Musca domestica* L. acetylcholinesterase in Danish housefly populations. Pest Management Science: Formerly Pesticide Science.

[ref-31] Li M, Reid WR, Zhang L, Scott JG, Gao X, Kristensen M, Liu N (2013). A whole transcriptomal linkage analysis of gene co-regulation in insecticide resistant house flies, *Musca domestica*. BMC Genomics.

[ref-32] Liang J, Xiao F, Ojo J, Chao WH, Ahmad B, Alam A, Abbas S, Abdelhafez MM, Rahman N, Khan KA, Ghramh HA, Ali J, Chen R (2025). Insect resistance to insecticides: causes, mechanisms, and exploring potential solutions. Archives of Insect Biochemistry and Physiology.

[ref-33] Litchfield JA, Wilcoxon F (1949). A simplified method of evaluating dose–effect experiments. Journal of Pharmacology and Experimental Therapeutics.

[ref-34] Liu N, Yue X (2000). Insecticide resistance and cross-resistance in the house fly (Diptera: Muscidae). Journal of Economic Entomology.

[ref-35] Lu X-P, Xu L, Wang J-J (2024). Mode of inheritance for pesticide resistance, importance and prevalence: a review. Pesticide Biochemistry and Physiology.

[ref-36] Ma Z, Li J, Zhang Y, Shan C, Gao X (2017). Inheritance mode and mechanisms of resistance to imidacloprid in the house fly *Musca domestica* (Diptera: Muscidae) from China. PLOS ONE.

[ref-37] Memmi BK (2010). Mortality and knockdown effects of imidacloprid and methomyl in house fly (*Musca domestica* L., Diptera: Muscidae) populations. Journal of Vector Ecology.

[ref-38] Mougabure-Cueto G, Picollo MI (2015). Insecticide resistance in vector Chagas disease: evolution, mechanisms and management. Acta Tropica.

[ref-39] Mu W, Wu K-M, Zhang W-J, Guo Y-Y (2004). Establishment of the near isogenic line strain esistant to lambda-cyhalothrin in *Spodoptera exigua* (Hübner)(Lepidoptera: Notuidae). Acata Entomologica Sinica.

[ref-40] Olagunju EA (2022). Housefly: Common zoonotic diseases transmitted and control. Journal of Zoonotic Diseases.

[ref-41] Onstad DW, Gassmann AJ (2023). Concepts and complexities of population genetics. Insect resistance management.

[ref-42] Pretty J, Bharucha Z (2015). Integrated pest management for sustainable intensification of agriculture in Asia and Africa. Insects.

[ref-43] Pu J, Chung H (2024). New and emerging mechanisms of insecticide resistance. Current Opinion in Insect Science.

[ref-44] Pu X, Yang Y, Wu S, Wu Y (2010). Characterisation of abamectin resistance in a field-evolved multiresistant population of *Plutella xylostella*. Pest Management Science: Formerly Pesticide Science.

[ref-45] Qian C, Li J, Wu S, Yang Y, Wu Y, Wang X (2024). Cross-resistance and genetics of field-evolved resistance to chlorfenapyr in *Plutella xylostella*. Insect Science.

[ref-46] Qu C, Yao J, Huang J, Che W, Fang Y, Luo C, Wang R (2024). Tetraniliprole resistance in field-collected populations of Tuta absoluta (Lepidoptera: Gelechiidae) from China: baseline susceptibility, cross-resistance, inheritance, and biochemical mechanism. Pesticide Biochemistry and Physiology.

[ref-47] Riaz B, Kashif Zahoor M, Malik K, Ahmad A, Majeed HN, Jabeen F, Zulhussnain M, Ranian K (2022). Frequency of pyrethroid insecticide resistance *kdr* gene and its associated enzyme modulation in housefly, *Musca domestica* L. populations from Jhang, Pakistan. Frontiers in Environmental Science.

[ref-48] Robertson JL, Jones MM, Olguin E, Alberts B (2017). Bioassays with arthropods.

[ref-49] Roush RT (1989). Designing resistance management programs: how can you choose?. Pesticide Science.

[ref-50] Roush RT, McKenzie JA (1987). Ecological genetics of insecticide and acaricide resistance. Annual Review of Entomology.

[ref-51] Sang S, Shu B, Yi X, Liu J, Hu M, Zhong G (2016). Cross-resistance and baseline susceptibility of Spodoptera litura (Fabricius) (Lepidoptera: Noctuidae) to cyantraniliprole in the south of China. Pest Management Science.

[ref-52] Sayyed AH, Attique MNR, Khaliq A, Wright DJ (2005). Inheritance of resistance and cross-resistance to deltamethrin in *Plutella xylostella* (Lepidoptera: Plutellidae) from Pakistan. Pest Management Science: Formerly Pesticide Science.

[ref-53] Sayyed A, Moore G, Crickmore N, Wright D (2008). Cross-resistance between Bacillus thuringiensis and non-Bt insecticides in *Plutella xylostella*. Pest Management Science.

[ref-54] Sayyed AH, Wright DJ (2006). Genetics and evidence for an esterase-associated mechanism of resistance to indoxacarb in a field population of diamondback moth (Lepidoptera: Plutellidae). Pest Management Science: Formerly Pesticide Science.

[ref-55] Schechtman H, Souza MO (2015). Costly inheritance and the persistence of insecticide resistance in Aedes aegypti populations. PLOS ONE.

[ref-56] Scott IM, Tolman JH, MacArthur DC (2015). Insecticide resistance and cross-resistance development in Colorado potato beetle Leptinotarsa decemlineata Say (Coleoptera: Chrysomelidae) populations in Canada 2008–2011. Pest Management Science.

[ref-57] Scott JG (2017). Evolution of resistance to pyrethroid insecticides in *Musca domestica*. Pest Management Science.

[ref-58] Shan C, Zhang Y, Ma Z, Gao X (2016). Inheritance of propoxur resistance in a near-isogenic line of *Musca domestica* (Diptera: Muscidae). Journal of Economic Entomology.

[ref-59] Shi J, Zhang L, Gao X (2011). Characterisation of spinosad resistance in the housefly *Musca domestica* (Diptera: Muscidae). Pest Management Science.

[ref-60] Shono T, Zhang L, Scott JG (2004). Indoxacarb resistance in the house fly, *Musca domestica*. Pesticide Biochemistry and Physiology.

[ref-61] Siddiqui JA, Fan R, Naz H, Bamisile BS, Hafeez M, Ghani MI, Wei Y, Xu Y, Chen X (2023). Insights into insecticide-resistance mechanisms in invasive species: Challenges and control strategies. Frontiers in Physiology.

[ref-62] Sokal RR, Rohlf FJ (1981). Biometry, third edition.

[ref-63] Spollen KM, Hoy MA (1992). Carbaryl resistance in a laboratory-selected strain of *Aphytis melinus* De Bach (Hymenoptera: Aphelinidae): mode of inheritance and implications for implementation in citrus IPM. Biological Control.

[ref-64] Stone B (1968). A formula for determining degree of dominance in cases of monofactorial inheritance of resistance to chemicals. Bulletin of the World Health Organization.

[ref-65] Tabashnik BE (1990). Modeling and evaluation of resistance management tactics. esticide Resistance in Arthropods.

[ref-66] Tian L, Cao C, He L, Li M, Zhang L, Zhang L, Liu H, Liu N (2011). Autosomal interactions and mechanisms of pyrethroid resistance in house flies, *Musca Domestica*. International Journal of Biological Sciences.

[ref-67] Tsukamoto M, Georghiou GP, Saito T (1983). Methods of genetic analysis of insecticide resistance. Pest resistance to pesticides.

[ref-68] Walsh T, Heckel DG, Wu Y, Downes S, Gordon K, Oakeshott J (2022). Determinants of insecticide resistance evolution: comparative analysis among Heliothines. Annual Review of Entomology.

[ref-69] White W, McCoy CM, Meyer JA, Winkle JR, Plummer PR, Kemper CJ, Starkey R, Snyder DE (2007). Knockdown and mortality comparisons among spinosad-, imidacloprid-, and methomyl-containing baits against susceptible *Musca domestica* (Diptera: Muscidae) under laboratory conditions. Journal of Economic Entomology.

[ref-70] Yıldırım İ, Çiftçi U (2022). Monitoring of pesticide residues in peppers from Çanakkale (Turkey) public market using QuEChERS method and LC–MS/MS and GC–MS/MS detection. Environmental Monitoring and Assessment.

[ref-71] Yoo J, Park C-G, Lee S-W, Choi B-R (2001). Cross resistance of cypermethrin-and methomyl-resistance and linkage group analysis on cypermethrin resistance in house fly (*Musca domestica* L.). Korean Journal of Applied Entomology.

[ref-72] Zhang X, Mao K, Liao X, Jiang T, Li J (2021). Inheritance mode and realized heritability of resistance to nitenpyram in the brown planthoper, Nilaparvata lugens Stål (Hemiptera: Delphacidae). Crop Protection.

[ref-73] Zhu X, Lei Y, Yang Y, Baxter SW, Li J, Wu Q, Wang S, Xie W, Guo Z, Fu W (2015). Construction and characterisation of near-isogenic *Plutella xylostella* (Lepidoptera: Plutellidae) strains resistant to Cry1Ac toxin. Pest Management Science.

